# Comparison of Two System Identification Approaches for a Four-Wheel Differential Robot Based on Velocity Command Execution

**DOI:** 10.3390/s25113553

**Published:** 2025-06-05

**Authors:** Diego Guffanti, Moisés Filiberto Mora Murillo, Marco Alejandro Hinojosa, Santiago Bustamante Sanchez, Javier Oswaldo Obregón Gutiérrez, Nelson Gutiérrez, Miguel Sánchez

**Affiliations:** 1 Universidad UTE, Av. Mariscal Sucre, Quito 170129, Ecuador; nelson.gutierrez@ute.edu.ec (N.G.); luis.sanchez@ute.edu.ec (M.S.); 2 Departamento de Mecánica y Ciencias Exactas, Instituto Superior Universitario Japón, Santo Domingo 230102, Ecuador; mmora@itsjapon.edu.ec; 3 Carrera de Electricidad, Instituto Superior Tecnológico Tsa’chila, Santo Domingo 230109, Ecuador; 4 Carrera de Electrónica, Instituto Superior Tecnológico Tsa’chila, Santo Domingo 230109, Ecuador; marcohinojosa@tsachila.edu.ec (M.A.H.); or javier.obregon@uleam.edu.ec (J.O.O.G.); 5 Carrera de Tecnología Superior en Mecánica Industrial, Instituto Superior Tecnológico Tsa’chila, Santo Domingo 230109, Ecuador; santiagobustamante@tsachila.edu.ec; 6 Universidad Laica Eloy Alfaro de Manabí sede Santo Domingo, Santo Domingo 230108, Ecuador

**Keywords:** differential robots, identification, motor-based model, simplified model, navigation

## Abstract

Precise modeling of differential drive robots is crucial for effective control and trajectory planning in autonomous systems. A comparative analysis of two modeling approaches for a four-wheel differential drive robot is presented in this paper. The first approach, named Motor-Based Model (MBM), identifies four transfer functions, one for each motor, while the second approach, named Simplified Model (SM), uses only two transfer functions, one for linear velocity and another for angular velocity. Both models were validated by comparing their predicted trajectories against real odometry data obtained from a SLAM system implemented on a differential-drive robot. This provided a practical assessment of each model’s accuracy and underscored the importance of model selection in control design and navigation tasks. The results showed that the Motor-Based Model (MBM) consistently outperformed the Simplified Model (SM) in terms of odometry accuracy, both in position and orientation. Across all trajectories, the average RMSE for position using MBM was 0.309 m, while the SM recorded a higher average RMSE of 0.414 m. Similarly, the maximum position error averaged 0.522 m for MBM and 0.710 m for SM, confirming that MBM is more accurate and consistent in position tracking. Regarding the results of orientation estimation, when averaged across all experiments, the MBM maintained a lower angular RMSE of 0.170 rad in contrast to SM, which achieves an RMSE of 0.239 rad. The maximum angular error was also higher for the MBM at 0.316 rad, compared to 0.447 rad for the SM. Moreover, the computational performance evaluation indicated that the SM consistently outperformed MBM, achieving a 30% reduction in simulation time and substantially lower memory usage. These results demonstrate the relationship between model complexity and accuracy and suggest that the motor-specific model is more appropriate for applications requiring precise mapping or localization, such as SLAM, while the simplified model may be suitable for simpler use cases with lower computational requirements, such as embedded systems with limited resources. This paper provides a practical evaluation of the accuracy and computational performance of two modeling approaches, highlighting the implications of model selection for the design of navigation tasks.

## 1. Introduction

In recent years, mobile robotics has experienced exponential growth, becoming a discipline of great importance in multiple sectors, ranging from manufacturing processes to space exploration [1]. Differential four-wheel drive robots have gained prominence due to their versatility, stability, and ability to perform precise maneuvers in a variety of operating environments [2]. This type of robot employs two or four independent motors to control each lateral pair of wheels, enabling more controlled and efficient mobility, making them ideal for applications requiring adaptability and precision.

A rigorous modeling process for this type of robot is essential for the design, control, and optimization of its performance [3]. Various modeling approaches consider both the kinematic and dynamic aspects of the traction system. Among these approaches, the most relevant are models based on direct and inverse kinematics, and those that address the dynamics of systems with multiple degrees of freedom. Each approach has its own advantages and limitations, which can significantly impact the efficiency of the control system and the robot’s response under varying operational conditions [4].

Although numerous studies have been conducted on the modeling and control of mobile robots, there is still a lack of comprehensive comparative analyses evaluating the performance of different modeling methods specifically applied to differential four-wheel drive robots [5]. This makes it difficult for researchers and engineers to select the most appropriate method to balance factors such as the complexity of the model, the required accuracy, and the robustness of the control system when designing a robot with this type of traction system [6].

This study presents a comparative analysis of two modeling approaches for a differential four-wheel drive robot. The first approach, known as the Motor-Based Model (MBM), involves obtaining a transfer function for each individual motor. By modeling each motor separately, the MBM aims to more accurately capture the robot’s behavior, particularly in terms of wheel movement. In contrast, the second approach, referred to in this study as the Simplified Model (SM), uses data from the robot’s Inertial Measurement Unit (IMU), which provides angular velocity measurements. The SM uses two transfer functions: one for the linear velocity based on encoder data and the other for the angular velocity derived from the IMU data.

Both approaches are velocity-based models; however, their effectiveness in accurately resolving the robot’s real-world odometry remains an open research question [7]. For this reason, the objective of this study is to analyze which of these modeling approaches is better at reproducing the robot’s odometry (x, y, θ) and which offers advantages in terms of computational efficiency. This will be achieved by adapting the predictions of the models to the calculation of simulated odometry data and comparing it with the real odometry obtained from a SLAM system, as well as by measuring simulation time and memory usage during repeated simulations. With the data obtained in this study, recommendations based on the results will be offered, thus contributing to the advancement of knowledge in the field of mobile robotics and facilitating the development of more efficient and adaptable robots.

This work aims to contribute in the following aspects: (1) since the approaches studied (MBM and SM) do not explicitly address dynamic modeling (forces, masses, torques, etc.), one of the important contributions lies in having avoided complexity by opting for a data-driven experimental approach, which is more direct, adaptable, and applicable in real environments. These models not only improve energy efficiency but also optimize the functionality of the robot in different applications. (2) Another contribution of this study is that it provides a realistic assessment of the performance of two modeling approaches for both position and orientation under varying motion trajectories using standard error metrics (RMSE, mean, standard deviation, and maximum error) and an evaluation of resource consumption of each, making clear the applications of each type of modeling approach. (3) Finally, the accuracy of the models was measured using a robotic system equipped with SLAM tools as a reference, which ensures the reliability of the results presented.

Based on these contributions, this study provides an important tool for deciding which type of modeling is the most suitable for specific applications and contributes to the advancement in the design and control of more efficient and autonomous robots, thus expanding the frontiers of robotic technology today.

The remainder of this paper is organized as follows: Section 2 presents a review of the state of the art in modeling and identification techniques for mobile robots. Section 3 describes the methodology, including the robotic platform used and the identification process for both the MBM and the SM approaches. Section 4 presents the comparative analysis and the accuracy of the odometry estimation achieved by each model. Section 5 discusses the results, comparing them with existing methods in the literature. Finally, Section 6 concludes this study and outlines directions for future work.

## 2. State-of-the-Art

Precise modeling of differential drive robots is essential for effective control and trajectory planning in autonomous systems. Various approaches have been proposed in the literature that focus on both kinematic and dynamic modeling techniques. Kinematic models describe the motion of robots without considering the forces that cause such motion. These models are fundamental for path-planning and control algorithms. For example, Bakirci et al. [8] present a comprehensive study on the kinematics, localization, and control of differential-drive mobile robots.

Dynamic models, on the other hand, take into account the forces and torques acting on the robot, providing a more detailed representation of its behavior. Dynamic equations are usually obtained using the Lagrange formulation or Newton–Euler-based methods [9]. This model is important for designing robust controllers that compensate for external disturbances [10]. Mohammed et al. [11] determine that it is necessary to analyze the dynamics of the automaton to limit the tracking error, especially in applications where high speed and load movements are taken into account. In the article, they develop an algorithm to optimize the internal parameters of the controller of a dynamic model based on a differential drive mobile robot; this one offers the advantages of fast response, high tracking accuracy, and excellent anti-interference capability.

Accurate parameter identification is critical to improving the reliability and predictive accuracy of robot models. Siwek et al. [12] detail the process of estimating dynamic parameters for two-wheeled robots with differential drive, highlighting how precise identification enhances the fidelity of the resulting models. This process enables the development of transfer function-based models, which provide a concise mathematical representation of the system’s input-output behavior and are particularly useful in control system design. For example, Ali et al. [13] propose a linear model predictive control framework combined with control barrier functions for differential drive robots, demonstrating how the transfer function of the identified models can be effectively applied to ensure safe and accurate navigation. In another paper, Tourajizadeh et al. [14] identified a parametric and numerical model of the motors of a robot. A method is proposed to control the torque and velocity of the motor simultaneously using the extracted dynamics of the motor, and consequently to control the robot motion more accurately. Test experiments showed that DC motor parameters can be affected by environmental conditions, so this model can produce errors in the control procedure as a result of parametric uncertainties and a non-linear effect. The identified parametric and numerical model was compared with a more current approach based on neural networks. The results demonstrated that the neural network model of the motor can cover all known and unknown motor parameters and is highly useful for the implementation of computer-based controllers.

Accurate model identification is essential for efficient control and trajectory tracking in differential drive robots. Although detailed motor-level models could provide high fidelity by capturing the dynamics of individual actuators, simplified models based on aggregate linear and angular velocities are often preferred for their ease of implementation and reduced complexity. However, it remains unclear how well these simplified representations approximate real robot behavior in practice and the effectiveness of each approach in resolving the robot’s real-world odometry. This paper addresses this gap by experimentally identifying both a Motor-Based Model (MBM), which includes one transfer function per motor, and a Simplified Model (SM), which uses only two transfer functions, one for linear velocity and another for angular velocity. Both models are validated by comparing their predicted trajectories with real odometry obtained from a SLAM system, providing a practical evaluation of their accuracy and highlighting the implications of model selection for control design and navigation tasks.

## 3. Methodology

### 3.1. The Differential Drive Robot

**Figure 1:**
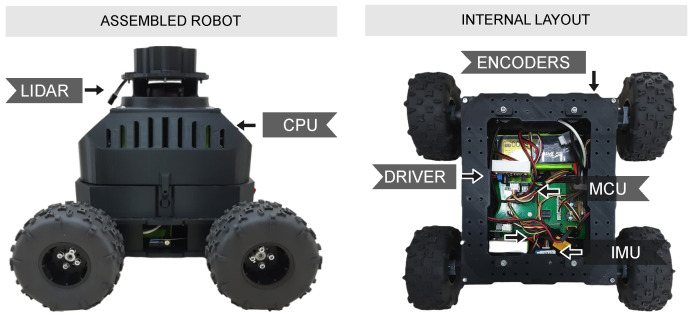
Real robot overview and instrumentation.

The mobile platform used in this study is a differential drive robot, with four wheels driven by four motors with encoders: forward left motor (FL), forward right motor (FR), rear left motor (RL), and rear right motor (RR). DC motors are 5203 Series Yellow Jacket Planetary Gear Motors from goBILDA, Winfield, USA (19.2:1 ratio, 312 RPM), each integrated with a quadrature encoder that provides a resolution of 537.7 pulses per revolution (PPR) at the output shaft. The motors and the rest of the robot structure are mounted on a Recon Chassis Kit, from the ServoCity company, Winfield, USA. The robot has two RoboClaw 2x15A motor controllers (drivers) from BasicMicro, Temecula, USA, associated with each left and right pair of motors. Drivers are responsible for controlling motor speeds based on commands received from an STM microcontroller (STM32-F412ZG), from STMicroelectronics, Geneva, Switzerland. The drivers have a built-in velocity controller that can be tuned through a user interface named Basicmicro Motion Studio. The controllers communicate with the microcontroller through a 38,400 baud USART serial connection. The microcontroller is connected to an onboard CPU through a serial port using a USB cable. The CPU runs the Robot Operating System 2 (ROS2), which is responsible for sending commands and receiving relevant data. The entire system has a sampling time of 0.0202 s, which corresponds to approximately 50 Hz. Additional components of the mobile robotic platform used in this paper can be seen in Figure 1. To support the reproducibility and technical transparency of the proposed methodology, Table 1 summarizes the main physical parameters of the mobile robot used in the experiments.

### 3.2. Model Identification

In the following subsections, we will describe in detail the two modeling approaches that are analyzed in this study, explaining their specific methodologies.

**Figure 2:**
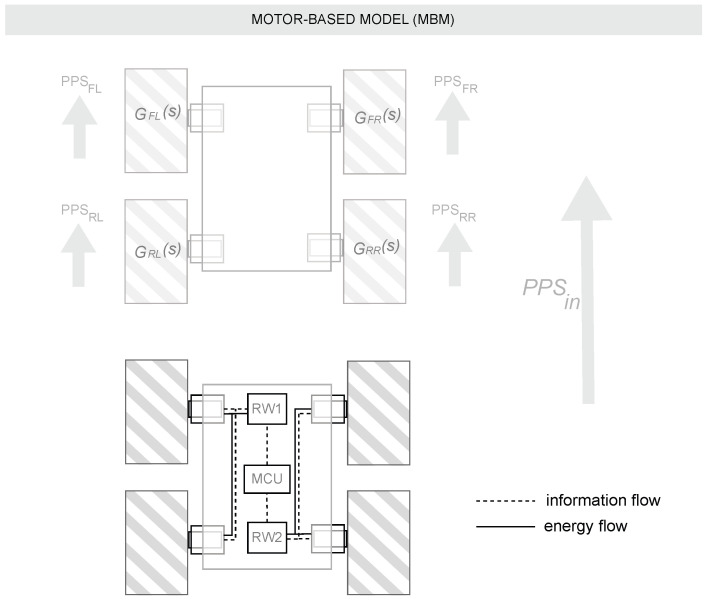
Experimental setup for the first modeling approach (MBM).

**Figure 3:**
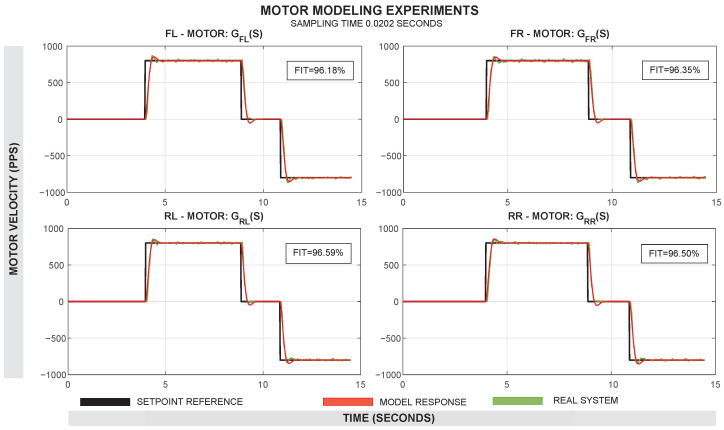
Comparison of the real motor response (green line) with the estimated model response (red line) for each motor. The input setpoint is shown for each case (black line).

**Figure 4:**
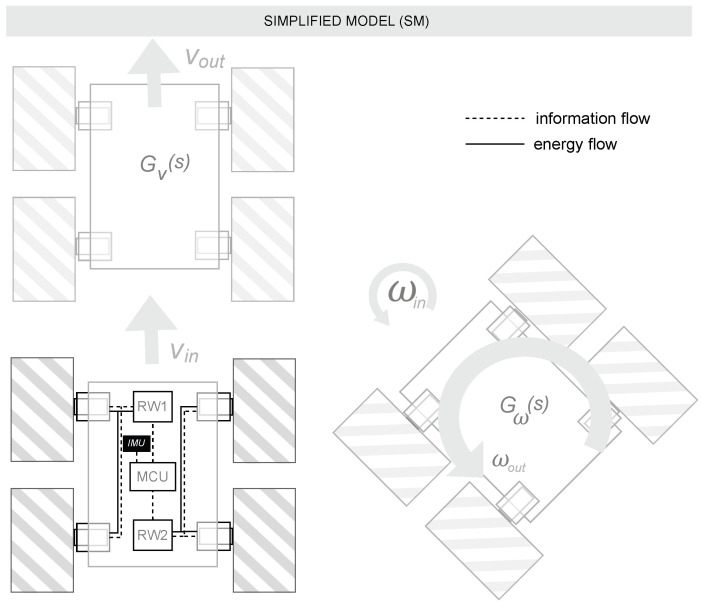
Experimental setup for the first modeling approach (SM).

**Figure 5:**
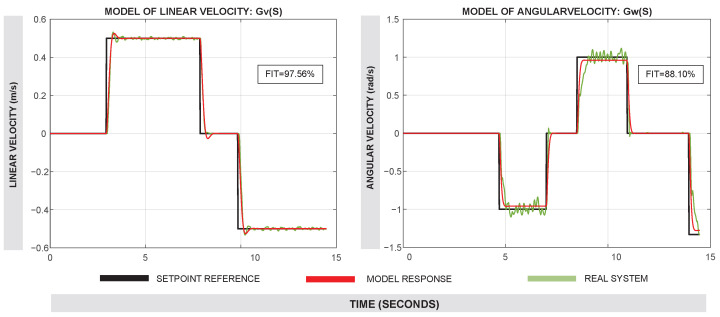
Comparison of real (red line) and estimated model response (green line) for $G_{v}$ and $G_{\omega }$. The input setpoint is shown in each case (black line).

- Motor-Based Model (MBM): The principle of this approach is based on modeling the behavior of each motor individually in order to know the behavior of the system as a whole using the kinematic equations of the differential robot. To perform this modeling, a total of four experiments were performed at 800, 1200, 1500, and 1800 pulses per second (PPS). The speed of motors in pps, derived from the encoder measurements and reported by the Roboclaw drivers, was collected. An illustration of this experimental setup can be seen in figure Figure 2.
  The four experiments per motor were merged into a single data object and processed using the System Identification Tool of MATLAB R2023b^®^. The model of each motor was estimated as a transfer function in the Laplace domain (s). The model was initially tested as a first-order transfer function with one pole and no zeros, but a second-order model with two poles and no zeros provided a better Fit Index (FIT). In this way, four transfer functions were obtained: $G_{FL}$, $G_{FR}$, $G_{RL}$, and $G_{RR}$. The resulting estimations had a Fit Index (FIT) greater than 90% in all motors. Default settings were used for model estimation, including the optimization algorithm (prediction error minimization with Gauss–Newton search) and validation (automatic data partitioning). A comparison between the real motor response and the identified model for each motor can be seen in Figure 3.
  The following transfer functions presents the identified models in the MBM approach:
  – Transfer function of the forward left motor $G_{FL}\left(s\right)$:
    (1) $$G_{FL}\left(s\right)=\frac{84.22}{s^{2}+12.01s+84.19}$$
  – Transfer function of the forward right motor $G_{FR}\left(s\right)$:
    (2) $$G_{FR}\left(s\right)=\frac{88.73}{s^{2}+12.49s+88.72}$$
  – Transfer function of the rear left motor $G_{RL}\left(s\right)$:
    (3) $$G_{RL}\left(s\right)=\frac{84.85}{s^{2}+12.35s+84.89}$$
  – Transfer function of the rear right motor $G_{RR}\left(s\right)$:
    (4) $$G_{RR}\left(s\right)=\frac{86.37}{s^{2}+12.16s+86.38}$$
  To use the predictions of these models in a subsequent calculation of the system’s odometry, it is necessary to calculate the linear velocity (*v*) and angular velocity (ω) of the system from these data. Using the predictions of these transfer functions, the PPS of each model is first transformed into angular velocities as follows:
  (5) $$\omega =\frac{PPS\cdot 2\pi }{e}$$
  where *e* is the encoder resolution in pulses per revolution and ω is the angular velocity of each motor in radians per second. For the right and left sides, the average angular velocities are calculated as follows:
  (6) $$\left[\begin{array}{c} \omega_{r}\\ \omega_{l} \end{array}\right]=\frac{1}{2}\left[\begin{array}{c} \omega_{fr}+\omega_{rr}\\ \omega_{fl}+\omega_{rl} \end{array}\right]$$
  Consequently, for a differential drive robot with wheel radius *r* (in meters) and distance *L* between the left and right wheels (in meters), the linear velocities of the right and left sides of the robot are given by the following:
  (7) $$\left[\begin{array}{c} v_{r}\\ v_{l} \end{array}\right]=r\cdot \left[\begin{array}{c} \omega_{r}\\ \omega_{l} \end{array}\right]$$
  Using this information, the overall linear and angular velocities of the robot are as follows:
  (8) $$\left[\begin{array}{c} v\\ \omega \end{array}\right]=\left[\begin{array}{c} \frac{v_{r}+v_{l}}{2}\\ \frac{v_{r}-v_{l}}{L} \end{array}\right]$$
- Simplified Model (SM): This second modeling approach relies on obtaining a model of the whole system, assisted by the data provided by the instrumentation itself. The term “Simplified” refers to the reduction in the number of equations or subsystems in this type of modeling. The system was equipped with an inertial measurement unit (IMU) that provides angular velocity data. The SM utilizes two transfer functions: one that estimates linear velocity (*v*) from encoder measurements, and another that estimates angular velocity (ω) from IMU readings. An illustration of this experimental setup can be seen in Figure 4.
  Four experiments were carried out using the real robot, testing at linear velocities of ±0.5 m/s and ±0.75 m/s. During these trials, the linear velocity (*v*) of the robot was collected. Using this information, a model for the linear velocity output from the linear velocity input $G_{v}$ was obtained. In another experiment, various angular velocities ranging from ±1.0 rad/s to ±1.5 rad/s were also tested, and the angular velocity from the IMU sensor of the robot was collected. This allowed us to obtain the model for the angular velocity output from the angular velocity input $G_{\omega }$.
  The experiments were merged into a single data object and processed using the System Identification Tool of MATLAB R2023b^®^. Second-order models with two poles and no zeros provided the best Fit Index (FIT), which in $G_{v}$ was 97.56% and in $G_{\omega }$ was 88.10%. Default settings were used for model estimation within the System Identification Tool of MATLAB. A comparison between the real system response and the identified models $G_{v}$ and $G_{\omega }$ can be seen in Figure 5.
  The following transfer functions present the identified models in the SM approach:
  – Transfer function of the linear velocity $G_{v}\left(s\right)$:
    (9) $$G_{v}\left(s\right)=\frac{109.7}{s^{2}+14.32s+109.9}$$
  – Transfer function of the angular velocity $G_{\omega }\left(s\right)$:
    (10) $$G_{\omega }\left(s\right)=\frac{346.8}{s^{2}+33.71s+361.7}$$

### 3.3. Odometry Calculation

This section focuses on explaining how to compute the robot’s odometry from the models’ predictions in both approaches analyzed in this study. For both modeling approaches, the way to obtain the linear velocity (*v*) and the angular velocity (ω) from the model predictions has been explained above. Afterwards, to calculate the odometry data, two different methods can be used, depending on the nature of the trajectory. In straight paths or for small angular velocities (ω), the robot’s displacement $\Delta x_{t}$ and $\Delta y_{t}$ for a given time period Δt can be accurately approximated using the simplified kinematic equations for linear motion in differential drive robots:

(11) $$\left[\begin{array}{c} \Delta x_{t}\\ \Delta y_{t}\\ \Delta \theta_{t} \end{array}\right]=\left[\begin{array}{c} v\cdot \cos\theta \cdot \Delta t\\ v\cdot \sin\theta \cdot \Delta t\\ \omega \cdot \Delta t \end{array}\right]$$

However, it tends to accumulate significant errors during curved trajectories. To address these limitations, this method is reinforced by considering the turning trajectory. When the robot has a non-zero angular velocity, its trajectory describes a circle whose center is known as the Instantaneous Center of Curvature (ICC), and whose radius is known as the turning radius *R*. The turning radius (*R*) allows the robot’s motion to be modeled as an arc to more accurately capture the true kinematics of differential drive robots during turns.

**Figure 6:**
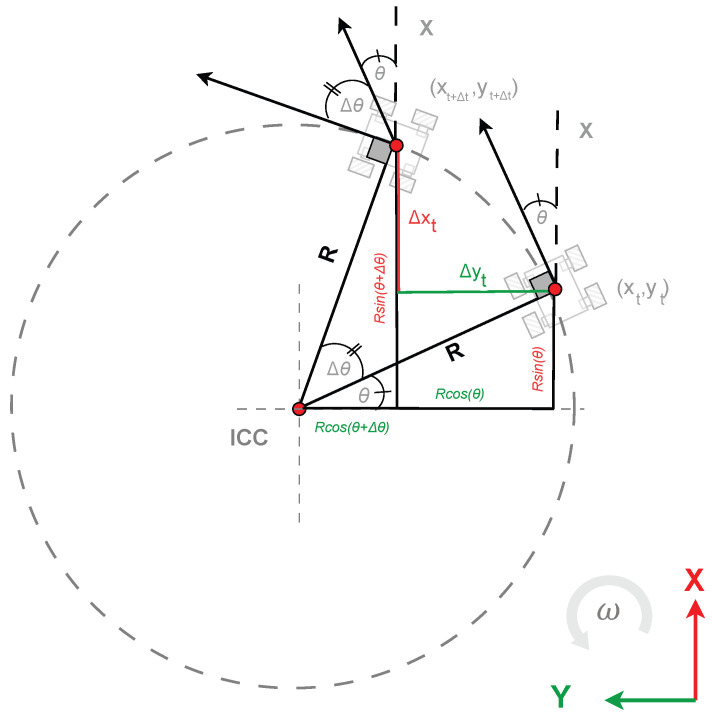
Relationships of circular motion for a robot moving in a trajectory with radius *R* and center in the ICC.

Using the relationships of circular motion in a trajectory with radius *R* and center in the ICC, as detailed in Figure 6, it is possible to calculate the displacement $\Delta x_{t}$ and $\Delta y_{t}$ of the robot for a given time period Δt, as follows:

(12) $$\left[\begin{array}{c} \Delta x_{t}\\ \Delta y_{t}\\ \Delta \theta_{t} \end{array}\right]=\left[\begin{array}{c} R\cdot \left(\sin(\theta +\omega \cdot \Delta t)-\sin(\theta )\right)\\ -R\cdot \left(\cos(\theta +\omega \cdot \Delta t)-\cos(\theta )\right)\\ \omega \cdot \Delta t \end{array}\right]$$

where the turning radius *R* is given by

(13) $$R=\frac{v}{\omega }$$

From there, the position and orientation of the robot can be updated as follows:

(14) $$\left[\begin{array}{c} x_{t+\Delta t}\\ y_{t+\Delta t}\\ \theta_{t+\Delta t} \end{array}\right]=\left[\begin{array}{c} x_{t}\\ y_{t}\\ \theta_{t} \end{array}\right]+\left[\begin{array}{c} \Delta x_{t}\\ \Delta y_{t}\\ \Delta \theta_{t} \end{array}\right]$$

where $x_{t}$, $y_{t}$, and $\theta_{t}$ represent the position and orientation at time *t*, and $\Delta x_{t}$, $\Delta y_{t}$, and $\Delta \theta_{t}$ are the increments calculated from the turning radius (*R*) and the angular velocity (ω) during the time step Δt.

### 3.4. Simulation and Testing

To validate both modeling approaches, the odometry predicted by each model was compared to the real robot’s odometry during experiments. The real robot’s odometry was estimated using a ROS2-based SLAM system, which integrates data from wheel encoders, an IMU, and a LiDAR. The Robot Operating System 2 (ROS2) is a powerful middleware for robot software development [15]. ROS2 Foxy Fitzroy version with the NAV2 package was used in the robotic system presented in this study. For the experimental phase, ROS2 was also used to publish the linear and angular velocity commands (*v*, ω) required to move the real robot. As mentioned above, the simulation of both modeling approaches was carried out using Simulink, within the MATLAB R2023b^®^ software environment.

**Figure 7:**
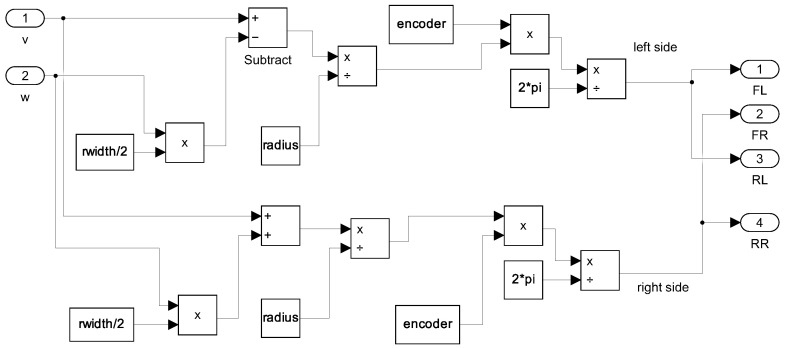
Block diagram to illustrate the transformation of velocity commands into PPS.

For the simulation of the MBM approach, it was necessary to transform these commands into PPS before introducing these setpoints into models $G_{FL}$, $G_{FR}$, $G_{RL}$, and $G_{RR}$. The transformation was carried out using a kinematic relation that maps the desired linear and angular velocities to the equivalent PPS values for each wheel, considering the wheel radius *r*, the distance between wheels *L*, and the encoder resolution *e*. The general form of this transformation is presented in Equation (15). A detailed representation of the implementation is provided in Figure 7.

(15) $$\left[\begin{array}{c} {\mathrm{PPS}}_{\mathrm{FL}}\\ {\mathrm{PPS}}_{\mathrm{FR}}\\ {\mathrm{PPS}}_{\mathrm{RL}}\\ {\mathrm{PPS}}_{\mathrm{RR}} \end{array}\right]=\frac{e}{2\pi }\cdot \left[\begin{array}{c} \frac{v}{r}-\frac{\omega L}{2r}\\ \frac{v}{r}+\frac{\omega L}{2r}\\ \frac{v}{r}-\frac{\omega L}{2r}\\ \frac{v}{r}+\frac{\omega L}{2r} \end{array}\right]$$

**Figure 8:**
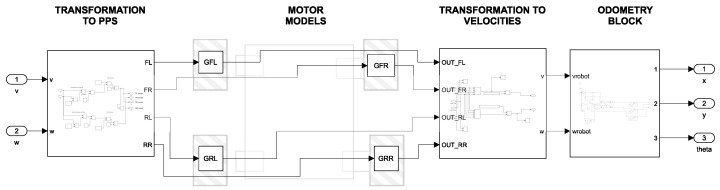
Simulink simulation diagram: Motor-Based Model (MBM).

Figure 8 shows the simulation of this first approach (MBM). Four stages can be observed in the simulation diagram. The first stage is a subsystem that serves for the transformation of the linear and angular velocity commands (*v*, ω) into PPS according to the transformation matrix shown in Equation (15). The second stage consists of the transfer functions of each motor: $G_{FL}$, $G_{FR}$, $G_{RL}$, and $G_{RR}$. The third stage is a subsystem that transforms the predictions of the transfer functions into linear and angular velocities (*v*, ω) according to the transformation matrices in Equations (5)–(8). Finally, the fourth stage is a subsystem to calculate the odometry of the robot and obtain the positions and orientation *x*, *y*, and θ, according to the transformation matrices from Equations (11)–(14).

**Figure 9:**
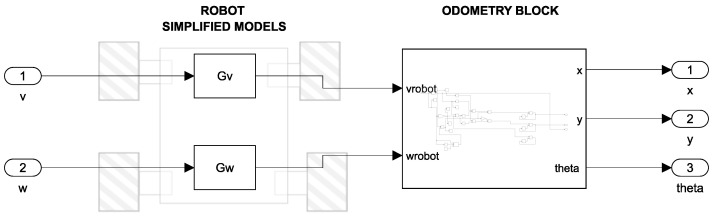
Simulink simulation diagram: Simplified Model (SM).

For the second modeling approach, the transformation of Equation (15) was not necessary since the model already provides the linear and angular velocity at the output and they can be introduced directly into the model, as shown in Figure 9. On this way, only two stages can be observed in Figure 9. The first stage contains the transfer functions of linear velocity ($G_{v}$) and angular velocity ($G_{\omega }$) while the second stage is a subsystem to calculate the odometry of the robot, according to the transformation matrices from Equations (11)–(14).

### 3.5. Trajectory Design and Validation

**Figure 10:**
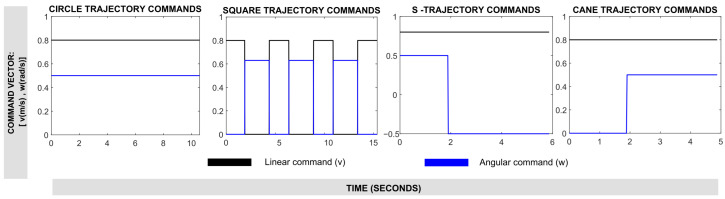
Experimental commands to generate robot motion trajectories.

Four experimental trajectories with different levels of complexity were developed for validation and comparison. Trajectories were defined based on linear and angular velocity commands (*v*, ω) with different durations for each, generating circular, square, S-shaped, and cane trajectories. The command combinations used for trajectory design are shown in Figure 10.

As mentioned above, in the real robot, these commands were published using the ROS2 framework. For this, a Python script was designed to publish the linear and angular velocity commands (*v*, ω) and their duration. The actual trajectory of the robot reported by the SLAM package was collected in a .csv file. This file contained the time stamp together with the published velocity commands. It also contained information about the robot’s odometry (x, y, θ) at each time stamp.

In order to ensure consistency between the simulation and real-world experiments, the same velocity command vector was applied to both. This approach guarantees that the command sequence is identical in the real system and across both modeling approaches, MBM and SM.

In addition, it is important to highlight that, although the identified models are linear transfer functions (second-order systems), no significant nonlinear effects such as those produced by motor saturation or dead zones are expected to be observed during the experiments. This is because the trajectories used for validation (circle, square, S-shaped, and cane trajectories) were executed with velocity commands (*v* and ω) that remained within the operational limits of the real robot. The microcontroller also enforces maximum limits for both linear and angular velocities, ensuring that the robot operates within a range where nonlinear effects are minimal.

**Figure 11:**
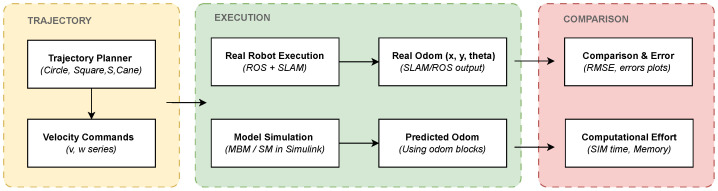
General block diagram for comparison.

Figure 11 summarizes the overall comparison process, illustrating the generation of velocity commands, their application to both the real robot and the simulation models, and the subsequent comparison between the real and predicted odometry. This diagram provides a structured overview of the methodology and highlights the interaction between trajectory planning, experimental and simulation execution, and error evaluation.

## 4. Results

This section presents the performance comparison between the Motor-Based Model (MBM) and the Simplified Model (SM) on four predefined trajectories: circle, square, S-shaped, and cane. The goal is to evaluate each model’s performance to predict real robot odometry, focusing on both position (x,y) and orientation (θ) estimation. The results are presented through graphical error maps and quantitative error metrics.

**Figure 12:**
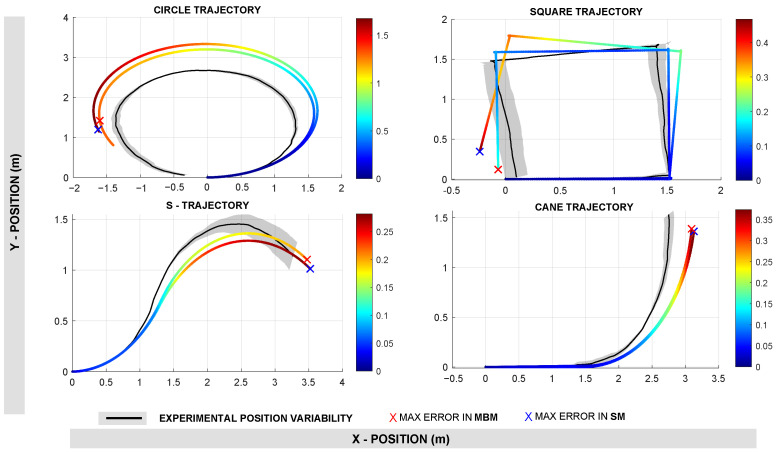
Results in robot position estimation. The color map shows the euclidean error (point-to-point) along the trajectory and the X marker shows the maximum error.

Figure 12 displays the position estimation performance for both approaches across all four trajectories. The color map represents the point-to-point Euclidean error, and the ‘X’ marker highlights the maximum error encountered during the trajectory. Visually, MBM generally shows lower error magnitudes in most trajectories. The quantitative results shown in Table 2 reinforce these observations. For example, on the circular trajectory, the MBM achieved a lower Root Mean Square Error (RMSE) of 0.848 m compared to 1.057 m for the SM. Similarly, for the square trajectory, the MBM had a significantly lower RMSE of 0.100 m versus 0.240 m for the SM. This pattern is consistent across the S-trajectory and the cane trajectory, where MBM outperformed SM with lower mean errors and standard deviations. Across all trajectories, the average RMSE for position using MBM was 0.309 m, while the SM recorded a higher average RMSE of 0.414 m. Similarly, the maximum position error averaged 0.522 m for MBM and 0.710 m for SM, confirming that MBM is more accurate and consistent in position tracking.

**Figure 13:**
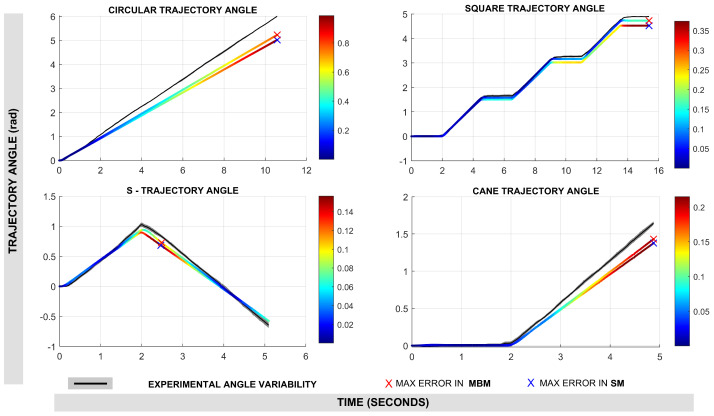
Results in robot angle estimation along the trajectory. The color map shows the euclidean error (point-to-point) along the trajectory and the X marker shows the maximum error.

Regarding the results of orientation estimation, Figure 13 illustrates the performance of both models in estimating robot orientation (θ). As with position, the color map denotes angular errors, and the maximum errors are marked with an ‘X’. Again, MBM generally shows lower angular discrepancies than SM. Table 2 also details the angular error metrics. In the circular trajectory, MBM achieved an RMSE of 0.452 rad, compared to 0.572 rad for the SM. The square trajectory shows a more pronounced difference, with MBM registering an RMSE of only 0.075 rad versus 0.184 rad for SM. Similar trends are observed in the S and cane trajectories. When averaged across all experiments, the MBM maintained a lower angular RMSE of 0.170 rad in contrast to SM, which achieves an RMSE of 0.239 rad. The maximum angular error was also higher for the MBM at 0.316 rad, compared to 0.447 rad for the SM.

### Simulation Performance Comparison

More than just comparing trajectory prediction accuracy, a computational performance comparison was conducted between the Motor-Based Model (MBM) and the Simplified Model (SM). Both models were simulated separately in MATLAB R2023b /Simulink using 100 independent runs each to compute the average and standard deviation statistics for key metrics over a trajectory of total simulation time, average time per step, and memory usage. To ensure a fair comparison, the memory was cleared between each simulation run by explicitly measuring the memory footprint before and after each execution. The MATLAB built-in function memory was used to track memory usage, while tic/toc measured the total simulation time. In addition, the average time per simulation step was calculated by dividing the total simulation time by the number of steps executed in each run. Each simulation consisted of 524 steps, which corresponded to one of the experimental trajectories used in the model comparison.

Table 3 summarizes the results obtained from 100 repetitions for each model. The SM consistently outperformed MBM in terms of computational efficiency, achieving a 30% reduction in simulation time and substantially lower memory usage. Specifically, the mean total simulation time was 0.158 s for the SM and 0.228 s for the MBM. The average computation time per step was also lower in the SM (0.00030 s) compared to the MBM (0.00043 s). In terms of memory, the SM required only 0.0053 MB on average, whereas the MBM consumed approximately 0.032 MB.

These performance gains are attributed to the reduced complexity of the SM, which models only two transfer functions corresponding to linear and angular velocity dynamics, in contrast to the MBM, which captures four separate motor dynamics. Moreover, the standard deviation values indicate that the MBM not only requires more resources but also exhibits higher variability across runs, likely due to the increased solver effort needed to handle its structure.

## 5. Discussion

The experimental results demonstrate that the Motor-Based Model (MBM) provides more accurate odometry estimation than the Simplified Model (SM) across all tested trajectories. This improved performance can be attributed to the fact that MBM captures the individual behavior of each motor through transfer functions derived from input-output data [16]. Although this does not represent the complete dynamics of the robot (e.g., inertial effects, wheel-ground interaction), it allows a more accurate approximation of actuator-level behavior, which directly affects the robot’s motion response. In contrast, SM relies on a data-driven approach that models the relationship between commanded and measured velocities, without explicitly considering the physical properties or internal dynamics of the robot.

This distinction highlights a fundamental trade-off in robotic system modeling. The MBM, by incorporating an experimental dynamics of each motor, could partially capture the internal system dynamics and therefore allows for better velocity prediction. This is consistent with findings from motor modeling research, such as [14], where accurate actuator models led to better motion control. Similarly, detailed identification of dynamic parameters has been shown to improve control performance in differential robots [12]; however, this process is more difficult to adjust, mainly due to the complexity of identifying dynamic parameters of the system.

In contrast, the SM is a simpler model that uses encoder data and IMU measurements to identify velocity transfer functions. Although this method is easy to implement and provides reasonably good performance for basic tasks, it may underperform in scenarios requiring high-precision motion, especially when nonlinear effects or disturbances are present. This aligns with observations in [17], where velocity integration alone was found to be insufficient for accurate localization without more sophisticated modeling or sensor fusion. Similar issues have been observed in other research focusing on visual odometry and machine learning for localization [18].

In addition, the fact that SM relies on IMU data introduces certain limitations. Although IMUs are useful for capturing angular velocity, their measurements can be subject to drift and noise (as was observed in Figure 5), which accumulate over time and reduce accuracy [13]. Without modeling how the robot responds physically to commands, the SM cannot fully compensate for these effects, leading to increased errors in odometry estimation. On the other hand, the MBM benefits from its closer alignment with the robot’s physical structure. Previous studies, such as [3,19], have emphasized the value of modeling the interaction between motor dynamics and robot kinematics, especially in differential drive configurations. Although our MBM does not explicitly model full-body dynamics as in [20], its improved odometry performance suggests that even partial modeling of system dynamics, focused at the actuator level, can be significantly beneficial.

A comparison of these modeling and control approaches is presented in Table 4. As can be seen in Table 4, our Motor-Based Model (MBM) achieved a position RMSE of 0.309 m and an orientation RMSE of 0.170 rad, outperforming the Simplified Model (SM) and aligning closely with other high-accuracy approaches. For example, the LG-based dynamic model [3] leverages Linear Graph theory and parameter estimation based on genetic algorithms to model a four-wheel skid steer robot. This approach achieved trajectory-tracking errors below 0.14 m in circular maneuvers and as low as 0.035 m in structured paths such as S-bends. While the LG model yields high fidelity and integrates multi-domain physical parameters, it requires complex offline calibration and a custom MATLAB-based modeling toolbox, resulting in moderate computational demands. In contrast, the Offset Differential model [19], while offering omnidirectional maneuverability, only presents qualitative validation without quantitative trajectory accuracy metrics, limiting its direct comparability. Meanwhile, the Identification of the dynamic parameters approach [12] demonstrates superior precision in controlled environments, achieving an RMSE of 0.009 m for position and 0.012 rad for orientation, but requires both offline and online identification procedures and exhibits a trajectory settling time of 7.5 s. These results suggest that, although MBM does not reach the sub-centimeter accuracy of closed-loop adaptive models, it offers a compelling balance of simplicity and accuracy for systems without high-end sensors or computational resources.

Furthermore, the results of this study highlight a clear trade-off between model accuracy and computational efficiency. The Motor-Based Model (MBM) delivers superior accuracy in both position and orientation tracking. This makes it particularly suitable for applications where precise localization is critical, such as SLAM-based mapping or high-fidelity trajectory tracking. However, this gain in accuracy comes at the cost of increased computational resources, as evidenced by higher simulation times and memory usage. On the other hand, the Simplified Model (SM), with its reduced complexity, demonstrates substantial gains in simulation speed and lower memory footprint. These characteristics suggest that the SM may be more appropriate for applications where computational constraints are stringent, such as embedded systems with limited resources, or in scenarios where approximate odometry is sufficient for navigation tasks. From a practical point of view, MBM may be more appropriate for applications that require accurate mapping or localization, such as SLAM, while the SM could be suitable for simpler use cases with lower computational requirements. This fits with the discussion in [5,21], where different levels of modeling fidelity were matched to different robot tasks and environments. In addition, models are the basis for control, such as Model Predictive Control (MPC), where a balance between accuracy and computational efficiency is sought [22,23]. A model such as MBM, reported as more accurate in our paper, allows the MPC to generate better predictions and make better decisions, especially in fast movements or near obstacles. In contrast, a model such as SM, while less accurate, can still be effectively used in MPC-based control schemes, particularly in applications where computational efficiency is prioritized over precision. Thanks to its lower computational cost, the SM approach is suitable for tasks with lower accuracy requirements, such as patrolling, cleaning, or simple point-to-point navigation in open areas.

## 6. Limitations and Practical Considerations

The comparison performed in this paper reveals that the basic motor-level modeling (MBM) can substantially improve odometry prediction compared to the black-box velocity modeling (SM). However, it is important to clarify that in this study, no experiments were performed under varying conditions, such as slippery surfaces or with additional payloads. Qualitatively, such conditions should cause errors primarily in translational and rotational motion estimations because linear models depend on parameters such as mass distribution and ground traction. For instance, on low-friction grounds, greater wheel slippage should result in underestimated displacement, whereas heavier payloads should result in slower acceleration responses that the existing linear dynamics fail to capture. Although this study does not perform a traditional sensitivity analysis, it is important to recognize that with extreme variations, the accuracy of the model would deteriorate. Future work may investigate methods for adaptive modeling or identification of non-linear systems that would perform better under such dynamic conditions.

The present study focuses on velocity-controlled odometry estimation, where inertial dynamics have a limited impact due to the moderate speeds and accelerations involved. For this reason, a full dynamic model of the robot was not considered in the comparative analysis. Both the motor-based model (MBM) and the simplified model (SM) capture relevant practical characteristics such as actuator delays and motor asymmetries from experimental data, which are commonly neglected in analytically derived dynamic models. While this choice favors implementation simplicity and real-time applicability, it also entails a trade-off in terms of generality. Future work could extend this framework by incorporating full dynamic models, particularly for tasks involving fast dynamics, external forces, or interaction with uneven terrain.

Furthermore, the modeling methods presented in this paper are scalable to robots with different wheel configurations or larger-scale platforms, considering only potential coupling effects (in MBM) or kinematic relationships (in SM). However, in the case of very heavy robots or robots with suspension systems (which cause typical effects such as roll or pitch) or six-wheeled robots (which are usually used to carry heavy loads), additional dynamic components should be added to the current models in order to incorporate these new dynamic behaviors and thus maintain accuracy.

Finally, in complex and extended trajectories, such as circular paths, odometry errors tend to accumulate due to physical factors like wheel slippage or drift. Additionally, motor parameters (e.g., resistance, inertia) and IMU biases can vary over time due to wear or temperature changes, potentially affecting long-term model performance. To address these issues, models intended for localization should be supported by external correction mechanisms (such as LiDAR or vision-based systems) that compensate for accumulated drift. Alternatively, the identified models are particularly well-suited for use in control strategies like Model Predictive Control (MPC), where their primary role is to provide short-term state prediction, while sensor fusion and state estimation techniques can handle drift, parameter variations, and external disturbances to ensure robust performance.

## 7. Conclusions

This study presented a comparative analysis of two modeling approaches for a differential four-wheel drive robot. The first approach, Motor-Based Model (MBM), modeled each motor separately to more accurately capture the robot’s behavior, particularly in terms of wheel movement. In contrast, the second approach, the Simplified Model (SM), used a reduced design to directly model input-output relationships in linear and angular velocities. The effectiveness of each approach in resolving the robot’s real-world odometry (x, y, θ) was analyzed. This was achieved by adapting the predictions of the models to the calculation of the simulated odometry data and comparing it with the real odometry obtained from a SLAM system. In summary, both graphical and numerical results indicated that the Motor-Based Model (MBM) consistently outperformed the Simplified Model (SM) in terms of odometry accuracy for both position and orientation. Across all trajectories, the averaged RMSE for position using MBM was 0.309 m, while the SM recorded a higher average RMSE of 0.414 m. Similarly, the maximum position error averaged 0.522 m for MBM and 0.710 m for SM, confirming that MBM is more accurate and consistent in position tracking. Regarding the results of orientation estimation, when averaged across all experiments, the MBM maintained a lower angular RMSE of 0.170 rad in contrast to SM, which achieves an RMSE of 0.239 rad. The maximum angular error was also higher for the MBM at 0.316 rad, compared to 0.447 rad for the SM. Although MBM does not explicitly model whole-body dynamics, its improved odometry performance suggests that even partial modeling of system dynamics, centered at the actuator level, can be significantly beneficial, as it involves closer alignment with the physical structure of the robot.

Moreover, the results of this study highlight a clear trade-off between model accuracy and computational efficiency. The evaluation of computational performance indicated that the SM consistently outperformed the MBM, achieving a 30% reduction in simulation time and substantially lower memory usage. This indicates that MBM is more accurate but has a higher computational cost while SM is less accurate but lighter. This leads to the conclusion that MBM may be more appropriate for applications requiring precise mapping or localization, such as SLAM, while SM may be suitable for simpler use cases with lower computational requirements, such as embedded systems with limited resources.

## Figures and Tables

**Table 1:** Main physical parameters of the mobile robot.

Parameter	Value	Description
Encoder resolution (e)	537.7 PPR	Pulses per revolution (PPR) at the output shaft
Radius (r)	0.0675 m	Robot wheel radius
Width (L)	0.330 m	Distance between left and right wheels
Weight (m)	6.850 kg	Robot weight including all components

**Table 2:** Odometry errors for MBM and SM methods.

	Position Errors (m)		Orientation Errors (rad)	
	MBM	SM	MBM	SM
**Circle Trajectory**				
RMSE	0.848	1.057	0.452	0.572
Mean Error	0.705	0.870	0.385	0.487
Standard Deviation	0.472	0.601	0.237	0.300
Max Error	1.311	1.675	0.776	0.988
**Square Trajectory**				
RMSE	0.100	0.240	0.075	0.184
Mean Error	0.091	0.203	0.057	0.146
Standard Deviation	0.042	0.127	0.049	0.113
Max Error	0.195	0.468	0.169	0.374
**S-Trajectory**				
RMSE	0.136	0.190	0.052	0.079
Mean Error	0.121	0.166	0.044	0.063
Standard Deviation	0.061	0.093	0.028	0.048
Max Error	0.208	0.282	0.105	0.156
**Cane Trajectory**				
RMSE	0.151	0.167	0.100	0.122
Mean Error	0.109	0.122	0.073	0.086
Standard Deviation	0.105	0.114	0.069	0.086
Max Error	0.373	0.416	0.215	0.269
**AVERAGE**				
RMSE	0.309	0.414	0.170	0.239
Mean Error	0.257	0.340	0.140	0.196
Standard Deviation	0.170	0.234	0.096	0.137
Max Error	0.522	0.710	0.316	0.447

**Table 3:** Simulation performance comparison between MBM and SM (100 runs).

Metric	MBM	SM
Sim Time Mean (s)	0.228	0.158
Sim Time Std (s)	0.116	0.023
Avg Time/Step Mean (s)	0.00043	0.00030
Avg Time/Step Std (s)	0.00022	0.000043
Mem Usage Mean (MB)	0.032	0.0053
Mem Usage Std (MB)	0.727	0.128

**Table 4:** Comparative analysis of modeling approaches. Several criteria are analyzed. For systems that do not study a certain criteria, the acronym N/A is used.

Criterion	MBM	SM	LG Model [3]	Offset D. [19]	Dyn. ID [12]
Description	Motor-specific, open-loop model	Velocity-based, open-loop model	Linear Graph-based dynamic model with GA-based ID	Omnidirectional control with offset wheels	Adaptive closed-loop model with parameter ID
Identification Accuracy	0.309 m/0.170 rad	0.414 m/0.239 rad	0.035–0.14 m (exp. traj. error)	Qualitative only	0.009 m/0.012 rad
Implementation	Moderate; per-motor ID	Low; simple model	High; LG toolbox + GA optimization	Moderate; custom geometry	Moderate; ROS tools + ID
Computation	High (0.00043 s/step, 32 MB)	Low (0.00030 s/step, 5.3 MB)	Moderate (≤0.1 s/step est.; MATLAB + GA)	Moderate	Moderate; reg. time 7.5 s
Physical Parameters	High	Low	High (mass, friction, geometry)	High (caster geometry)	High (mass, friction, etc.)
Generalizability	Medium	High	Medium-High; applicable to similar wheeled robots	Low	Medium
Applications and HW	SLAM; 4 encoders	Embedded nav; 2 encoders	ROS/Gazebo; IMU, encoders, MATLAB	Indoor nav; 3 motors, caster	AGV; IMU, ROS, torque access
Tracking Time	N/A	N/A	Not reported	Not reported	7.5 s (8-shape)

## Data Availability

Dataset available on request from the authors.
